# A Case of Rapidly Progressive Pneumonitis Induced by Nivolumab in Metastatic Gastroesophageal Adenocarcinoma: Challenges in Diagnosis and Management

**DOI:** 10.7759/cureus.94853

**Published:** 2025-10-18

**Authors:** Nitish Datta, Ana C Vieira, Catherine Strait

**Affiliations:** 1 Medical Education/Acute Medicine, Great Western Hospital, Swindon, GBR; 2 Internal Medicine, Great Western Hospital, Swindon, GBR; 3 Acute Medicine, Great Western Hospital, Swindon, GBR

**Keywords:** cancer immunotherapy, drug induced pneumonitis, immune-checkpoint inhibitors, immune-mediated pneumonitis, immune-related adverse events (iraes), nivolumab-related adverse events, oncology care, pd-l1 inhibitors, programmed cell death protein 1 pathway inhibitors, rapid clinical deterioration

## Abstract

Immune checkpoint inhibitors like nivolumab have transformed cancer therapy but are associated with immune-related, sometimes life-threatening, adverse events, including pneumonitis. We report a case of a 55-year-old man with metastatic gastroesophageal adenocarcinoma who developed rapidly progressive pneumonitis within a week of initiating nivolumab and FOLFOX therapy (folinic acid, 5-fluorouracil and oxaliplatin). Despite some initial improvement with antibiotics and ongoing oral steroids, the patient deteriorated rapidly and died. This case highlights the importance of early recognition and aggressive management of immune-related pneumonitis. Clinicians must be aware of such adverse events, maintain a high index of suspicion and adopt a multidisciplinary approach to optimize outcomes, especially given the often subtle and rapidly evolving clinical presentation.

## Introduction

Immune checkpoint blockade therapies targeting the programmed cell death protein 1/programmed death ligand 1 (PD-1/PD-L1) axis are now FDA-approved for treating a wide variety of tumours and are likely to be approved for additional indications in the near future [1]. However, there is emerging evidence of autoimmune conditions such as type 1 diabetes, fulminant myocarditis and pneumonitis developing as potential immune-related adverse events (irAEs) due to anti-PD-1 therapy [2,3]. These toxicities are thought to result from the inhibition of the PD-1/PD-L1 pathway, which normally suppresses autoreactive T-cells. This disruption can lead to uncontrolled T-cell activation and autoimmune responses, causing inflammation and damage to various organs. Nivolumab, a human IgG4 monoclonal antibody that acts against PD-1 and blocks a co-inhibitory signal on PD-1/PD-L1 and PD-1/PD-L2 axis, is emerging as a novel treatment strategy for advanced gastric cancers [4,5]. Certain studies have shown the occurrence of nivolumab treatment-related adverse events, including deaths; however, these were also partly attributed to already existing illnesses in patients [5]. Among these serious adverse events, pneumonitis is one of the most life-threatening occurrences, with a variety of presentations and image patterns on chest X-rays [6]. The time to onset of pneumonitis induced by these immune checkpoint blockade therapies shows considerable variability, ranging from 9 days to 19.2 months [7]. Pneumonitis induced by nivolumab, particularly with cryptogenic organizing pneumonia or non-specific interstitial pneumonia patterns, has been shown to have increased lethality, as a result of diffuse alveolar damage [7-9]. While nivolumab increases the overall survival in advanced gastric cancer patients, it is associated with severe adverse effects, including pneumonitis, thus warranting careful monitoring to strike a proper balance between efficacy and safety [10]. We present a case where a patient having metastatic gastroesophageal adenocarcinoma was started on treatment with FOLFOX therapy (folinic acid, fluorouracil and oxaliplatin) and nivolumab and developed pneumonitis within a week of starting treatment. He presented with symptoms indicative of a likely chest infection, with symptoms initially improving with antibiotic treatment. This was, however, followed by a rapid deterioration in health within 36 hours, which proved to be fatal. The aim of this case report is to highlight the potential for early-onset immune-related pneumonitis following immune checkpoint inhibitor therapy and the diagnostic challenges due to its non-specific presentation. This report also emphasizes the importance of early recognition and multidisciplinary management to improve patient outcomes.

## Case presentation

**Figure 1:**
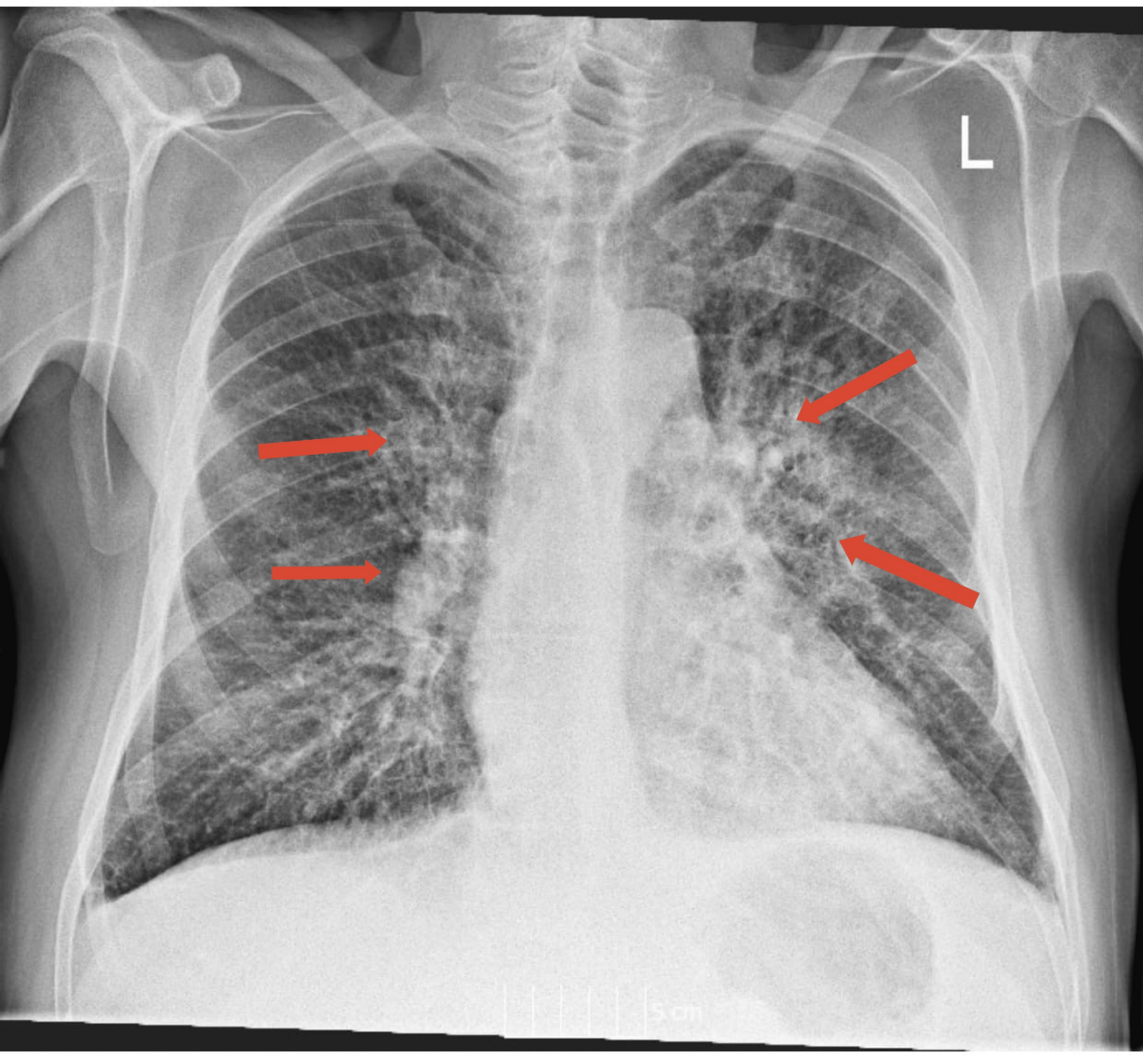
Chest X-ray taken at the time of hospital presentation. Chest X-ray taken at the time of hospital presentation, showing new bilateral diffuse perihilar inflammatory changes (indicated by arrows).

A 55-year-old man presented to the emergency department with progressive shortness of breath and a mild non-productive cough. He was diagnosed one month ago with adenocarcinoma of the gastroesophageal junction at the stomach, with extensive liver metastasis. The patient’s past medical history only included bilateral small pulmonary emboli diagnosed approximately two weeks prior to the cancer diagnosis, for which he was receiving a direct oral anticoagulant (DOAC). His cancer was found to be HER2 (human epidermal growth factor receptor 2) negative and PDL1 positive for FOLFOX and nivolumab, and the patient had received the first cycle of this therapy one week before the date of presentation to the emergency department. The patient had also received a single dose of radiotherapy about one month back and was on dexamethasone 4mg once a day, since the last two and a half weeks, prescribed by the Oncologist. The patient had a National Early Warning Score (NEWS2) of 4, with a peripheral oxygen saturation (SpO2) of 92% while receiving 4 litres of oxygen. The patient’s white cell count (WBC) had a higher-than-normal baseline, around 20,000 cells per microliter (μL), possibly due to the ongoing inflammatory response from the cancer itself and aggravated using dexamethasone and the single dose of radiotherapy. The C-reactive protein (CRP) had a raised value of 155 mg/L (baseline CRP for the patient was 110mg/L). The chest X-ray showed new bilateral diffuse perihilar inflammatory changes (Figure 1). 

**Figure 2:**
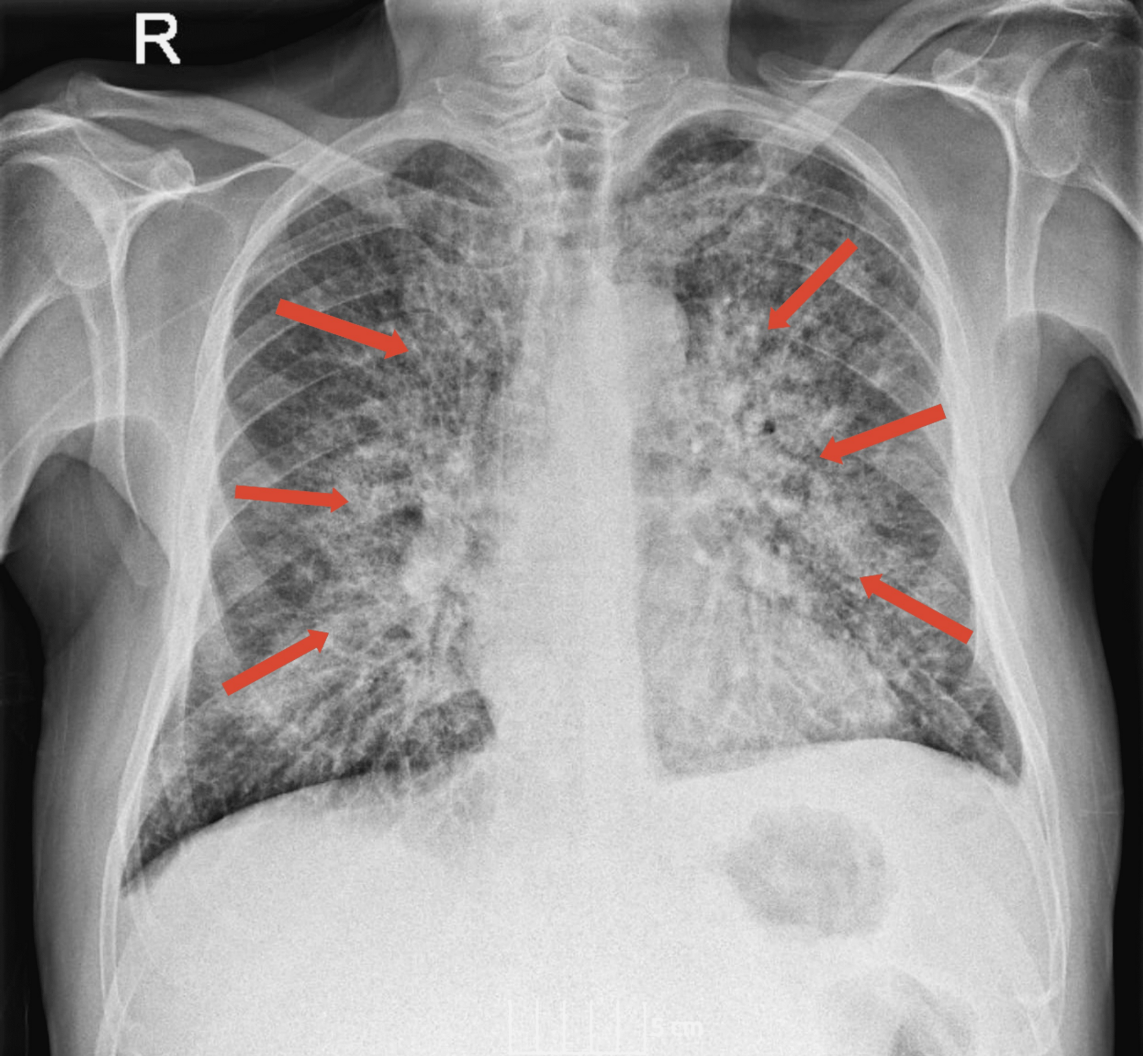
Repeat chest X-ray (48 hours after hospital admission). Repeat chest X-ray (48 hours after hospital admission), showing increase in the prominence of perihilar shadowing in the lungs (indicated by arrows).

The patient was treated with intravenous co-amoxiclav antibiotics, while the dexamethasone dose was doubled following sick day rules of steroid treatment. The patient showed significant clinical improvement, and after forty-eight hours, had a SpO2 of 95% on room air. However, repeat blood testing showed no significant improvement in the CRP value. A repeat chest X-ray showed an increase in the prominence of perihilar shadowing in the lungs (Figure 2). 

The patient was switched to treatment with oral co-amoxiclav. Thirty-six hours later, the patient’s condition suddenly deteriorated, with his SpO2 falling to 93% while receiving 5 litres of oxygen and a lactate value of 4.2. Following the advice of the oncologist and radiologist, the patient was started on intravenous methylprednisolone. Both specialists believed that the changes observed in the repeat chest X-ray occurred too rapidly to be tumour-related lymphangitis and were more likely to represent autoimmune pneumonitis. The patient’s condition deteriorated further over the next 10 hours, with a continued drop in SpO2 requiring 15 litres of oxygen. The patient subsequently passed away.

## Discussion

Nivolumab disrupts PD-1 and PD-1 ligand 1/2 (PD-L1/2) interaction, which plays a crucial role in cancer immune tolerance, thus restoring the antitumour activity of tumour-specific T-cells [11]. The clinical usage of nivolumab has rapidly increased due to various clinical studies showing its benefits in the treatment of different advanced cancers [12,13]. However, nivolumab use is also associated with various adverse effects, including lung diseases like infectious pneumonia and immune-related pneumonitis, with the latter usually being rapidly progressive [14]. One study showed the occurrence of pneumonitis in 43 out of 915 patients who received anti-PD-1/PD-L1 monoclonal antibodies [7]. This case report highlights the importance of recognizing immune-related pneumonitis as a rare but serious adverse effect of nivolumab therapy, having the potential to cause rapid clinical deterioration despite initial apparent improvement. Immune-checkpoint inhibitor-associated pneumonitis can have life-threatening flares while presenting with non-specific symptoms and ambiguous radiographic findings [15]. Some conditions which resemble the presentation of nivolumab-related pneumonitis include pneumonia, tumour progression, immune-related tumour inflammation, pulmonary oedema and alveolar haemorrhage [16,17]. Due to the subtle nature of the symptoms, a thorough evaluation of the patient is essential to avoid delays or missing the correct diagnosis [18]. Clinicians should have a low threshold for performing CT scans in such patients, to look for features unique to nivolumab-associated pneumonitis rather than focusing on conventional drug-induced pneumonitis characteristics [19]. The radiographic features in such cases can rapidly evolve from a pattern of low-grade treatment-related pneumonitis to an acute respiratory distress syndrome (ARDS) pattern of disease [15]. To prevent such a life-threatening adverse effect and facilitate timely therapeutic intervention, vigilant monitoring of patients on nivolumab therapy is recommended [20]. This includes close monitoring of new or worsening respiratory symptoms, oxygen saturation levels, inflammatory markers such as CRP and WBC, and early use of imaging, particularly high-resolution CT scans, when clinically warranted. Due to the severity of symptoms not always relating to the radiological findings or the actual severity of the condition, it is highly recommended to involve a multidisciplinary team to improve clinical management [21]. Despite the presence of established treatment guidelines, it is essential to involve consultants from various specialities - infectious disease, respiratory and oncology, early, to determine an appropriate, comprehensive, case-specific treatment plan, especially for complicated cases [18]. The management of immune-related adverse events in patients treated with immune-checkpoint inhibitor therapy generally involves suspension of the causative immunotherapy drug and initiation of corticosteroids [22]. Most patients show improvement upon treatment with high-dose corticosteroids; however, one case series showed the worsening of 12% of patients with grade 3 or higher pneumonitis while being treated with corticosteroids, and despite additional immunosuppression, the patients ultimately died [7]. Although this case report has the limitation of showing the occurrence of severe pneumonitis in only one patient, there are various studies on different patients showing the high incidence of nivolumab-related pneumonitis with an early onset [6]. Due to a lack of histopathology reports, it is difficult to conclusively prove that the occurrence of autoimmune pneumonitis in this case was due to nivolumab, as other factors, such as radiotherapy when combined with immunotherapy, have also been shown to cause adverse effects like pneumonitis [23]. However, radiotherapy-induced pneumonitis generally develops as a result of radiotherapy being delivered directly to the lungs [24] rather than other organs. In our case report, radiotherapy was delivered to the gastroesophageal region of the stomach. Other factors indicating the pneumonitis to have occurred as an adverse effect of nivolumab therapy include the absence of any new drugs being initiated in the period between nivolumab initiation and the occurrence of pneumonitis, and broad-spectrum antibiotics being ineffective in managing the condition [14]. The prior administration of dexamethasone and radiotherapy raises the possibility that the lung milieu was already primed for exaggerated immune injury. This case report also underscores the importance of having more comprehensive risk-benefit assessments while prescribing immune checkpoint inhibitors like nivolumab [25].

## Conclusions

Due to the high incidence of immune-related adverse events associated with immune checkpoint inhibitors, it is important for clinicians to be thoroughly familiar with these potential complications and remain vigilant when prescribing such medications. Immune-related pneumonitis, though rare, can rapidly progress and end up being life-threatening. It may present with nonspecific symptoms, making early detection and intervention critical. This case emphasizes the need for careful monitoring and a multidisciplinary approach to manage these patients effectively. More research is needed to identify patients at greater risk of developing nivolumab-associated pneumonitis and to understand how co-factors, such as prior therapies like radiotherapy, may interact with immunotherapy to trigger such complications. Expanding our understanding of these immune checkpoint inhibitor-related adverse events will help guide appropriate treatment strategies for emerging complications in cancer therapy.

## References

[1] Wei SC, Duffy CR, Allison JP (2018). Fundamental mechanisms of immune checkpoint blockade therapy. Cancer Discov.

[2] Johnson DB, Balko JM, Compton ML (2016). Fulminant myocarditis with combination immune checkpoint blockade. N Engl J Med.

[3] Moslehi JJ, Salem JE, Sosman JA, Lebrun-Vignes B, Johnson DB (2018). Increased reporting of fatal immune checkpoint inhibitor-associated myocarditis. Lancet.

[4] Kono K, Nakajima S, Mimura K (2020). Current status of immune checkpoint inhibitors for gastric cancer. Gastric Cancer.

[5] Kang YK, Boku N, Satoh T (2017). Nivolumab in patients with advanced gastric or gastro-oesophageal junction cancer refractory to, or intolerant of, at least two previous chemotherapy regimens (ONO-4538-12, ATTRACTION-2): a randomised, double-blind, placebo-controlled, phase 3 trial. Lancet.

[6] Koyama N, Iwase O, Nakashima E (2018). High incidence and early onset of nivolumab-induced pneumonitis: four case reports and literature review. BMC Pulm Med.

[7] Naidoo J, Wang X, Woo KM (2017). Pneumonitis in patients treated with anti-programmed death-1/programmed death ligand 1 therapy. J Clin Oncol.

[8] Kato T, Masuda N, Nakanishi Y (2017). Nivolumab-induced interstitial lung disease analysis of two phase II studies patients with recurrent or advanced non-small-cell lung cancer. Lung Cancer.

[9] Koelzer VH, Rothschild SI, Zihler D (2016). Systemic inflammation in a melanoma patient treated with immune checkpoint inhibitors-an autopsy study. J Immunother Cancer.

[10] Lei X, Huo W, Xu T, Xu J, Liu M, Liu C, Gu Z (2024). Efficacy and safety of nivolumab in advanced gastric and gastroesophageal junction cancer: a meta-analysis of randomized controlled trials. BMC Gastroenterol.

[11] Okazaki T, Chikuma S, Iwai Y, Fagarasan S, Honjo T (2013). A rheostat for immune responses: the unique properties of PD-1 and their advantages for clinical application. Nat Immunol.

[12] Robert C, Long GV, Brady B (2015). Nivolumab in previously untreated melanoma without BRAF mutation. N Engl J Med.

[13] Borghaei H, Paz-Ares L, Horn L (2015). Nivolumab versus docetaxel in advanced nonsquamous non-small-cell lung cancer. N Engl J Med.

[14] Tada K, Kurihara Y, Myojo T (2017). Case report of nivolumab-related pneumonitis. Immunotherapy.

[15] Shea M, Rangachari D, Hallowell RW, Hollie NI, Costa DB, VanderLaan PA (2018). Radiologic and autopsy findings in a case of fatal immune checkpoint inhibitor-associated pneumonitis. Cancer Treat Res Commun.

[16] Watanabe S, Kimura H, Takato H (2016). Severe pneumonitis after nivolumab treatment in a patient with melanoma. Allergol Int.

[17] Chow LQ (2013). Exploring novel immune-related toxicities and endpoints with immune-checkpoint inhibitors in non-small cell lung cancer. Am Soc Clin Oncol Educ Book.

[18] Li C, Faiz SA, Boysen-Osborn M, Sheshadri A, Wattana MK (2025). Immune checkpoint inhibitor-associated pneumonitis: A narrative review. West J Emerg Med.

[19] Baba T, Sakai F, Kato T (2019). Radiologic features of pneumonitis associated with nivolumab in non-small-cell lung cancer and malignant melanoma. Future Oncol.

[20] Sumi T, Sekikawa M, Koshino Y (2024). Risk factors for severe immune-related pneumonitis after nivolumab plus ipilimumab therapy for non-small cell lung cancer. Thorac Cancer.

[21] Valente M, Colucci M, Vegni V (2024). A multidisciplinary approach to improve the management of immune-checkpoint inhibitor-related pneumonitis. Onco Targets Ther.

[22] Brahmer JR, Lacchetti C, Schneider BJ (2018). Management of immune-related adverse events in patients treated with immune checkpoint inhibitor therapy: American society of clinical oncology clinical practice guideline. J Clin Oncol.

[23] Zhang A, Yang F, Gao L, Shi X, Yang J (2022). Research progress on radiotherapy combined with immunotherapy for associated pneumonitis during treatment of non-small cell lung cancer. Cancer Manag Res.

[24] Keffer S, Guy CL, Weiss E (2020). Fatal radiation pneumonitis: literature review and case series. Adv Radiat Oncol.

[25] Wu Y, Zhou Y, Xia S, Meng Z (2025). The real-world safety of Nivolumab: a pharmacovigilance analysis based on the FDA adverse event reporting system. Front Immunol.

